# Mean field game model of the impact of reductions in support on faculty research activity

**DOI:** 10.1073/pnas.2538029123

**Published:** 2026-06-02

**Authors:** Robert A. Brown

**Affiliations:** ^a^ Faculty of Computing & Data Sciences, Boston University, Boston, MA 02215

**Keywords:** mean field game theory, research universities, numerical solution, faculty research

## Abstract

Every research university has a distribution of faculty research activity that is fueled by the external support from government, industry, and philanthropy. The large decreases in federal support proposed in 2025 by the Administration would dramatically alter the distribution of faculty research activity. Mean field game (MFG) theory is used to model federal support of faculty research assuming that each researcher seeks to maximize their funding according to a specified utility function. Modeling the collection of researchers as a continuum with individual researchers only influenced by the distribution of others—referred to as the mean field—predicts ergodic distributions that represent a Nash equilibrium. Results representing the funding for faculty in colleges of engineering demonstrate that a decrease in overall federal support of the magnitude proposed by the Administration could lead to over half the faculty inadequately funded to support research. The results have important implications to the university research environment and to their strategies for maintaining the vibrancy of their programs.

## Significance

The vibrancy of United States university science and engineering research depends on the competitive interactions of research faculty who win federal grants for support. Mean field game theory gives a framework for understanding the impact of proposed funding cuts and of actions that bias the system on the health of research universities. The predictions are the basis for strategies for preserving these institutions.

For 75 y, research in universities in the United States has been fueled by federal support through grants to faculty mostly distributed through a peer review system. Faculty researchers submit proposals that are reviewed by their peers with the most meritorious receiving support. With the creation of the NSF in 1950 and spreading to other agencies, most importantly the NIH, this system embedded the teacher-scholar model for faculty in research universities, where active researchers teach undergraduates. The peer review system also democratized research support, funding faculty across the university system. In 2025, 174 public and private universities (not counting stand-alone medical schools) were categorized as R1—very research intensive.

Today, the future of this system is in question with the traditionally bipartisan federal support wavering and proposals from the Administration to dramatically cut research funding and to tie grants to adherence to political agendas instead of merit. Even before the current Administration began attacking research universities, there were cracks forming in the system. Most notable was the large and growing portion of university research funded by the institutions; in 2022 the R1’s spent 46 cents for every dollar of federal support they received (1). It was unclear whether universities could maintain this level of support while meeting challenges caused by demographic shifts, the pressures for cost control, and the need to increase student access and improve outcomes. This was the case before the Trump Administration’s attempt to decimate the system. Although the congressional research budget for 2026 rejected most of the proposed cuts, the concerns are heightened about the sustainability of the American research university.

The research activity within each university is driven by the faculty who propose and supervise the research conducted by doctoral students and postdoctoral researchers. I focus on STEMM (science, technology, engineering, mathematics, and medicine) fields where external funding for supporting people and laboratories is essential to the research enterprise. These faculty are members of academic departments that typically administer doctoral programs where external funding is critical for student support.

In every research university, the research activity of individual faculty varies, either because of the stages of faculty careers, their interest, or ability to acquire external funding. Some faculty who were once research active become inactive, with their support level falling below a minimum threshold needed to maintain their effort. In other cases, faculty are hired without the expectation of being research active, predominately to teach undergraduates or in specialized graduate programs. Usually, but not always, the titles of these faculty differ from the research active faculty on the tenure-track.

If federal support decreases, an important question is what will happen to the distribution of research active faculty within each department and university? In a previous study (2), data from Boston University and from colleges of engineering, as well as a simple probabilistic model was used, to estimate a significant increase in the fraction of research inactive faculty if federal support decreases precipitously. The argument relied on correlating the stochastic model with research expenditure data for colleges of engineering and using parametric variation to predict the impact of research decreases on the faculty distribution.

The analysis here takes another approach by developing a mean field game (MFG) model for research funding. Originated by Lasry and Lions (3–5) and in parallel by Huang et al. (6) mean field game theory was developed for the case of a large number of agents (here researchers) and combines stochastic optimal control with a mean field approximation for a distribution of agents to predict the optimal state of an agent as a function of the actions of the mean field. The importance of the continuum MFG theory results from its link to the results for a finite number of agents, where the well-known Nash equilibrium represents the state where no agent would gain more by changing their strategy if the strategies of all the other agents remain constant (7). Assuming that the strategies of all agents in a mean field population have the same form, the distribution predicted by the MFG approximates the Nash distribution for a finite population as the number of agents becomes large. In the context of the distribution of research funding among researchers, the solution of the MFG model gives a framework for modeling the impact of changing federal policies or other external shocks.

Mathematically, the MFG theory results in two coupled nonlinear partial differential equations described in terms of time and a continuous state variable representing researcher effectiveness. The equations are a Hamilton–Jacobi–Bellman equation for the optimal control problem and a Fokker–Planck–Kolmogorov equation for the distribution of researchers. We solve these equations in the ergodic limit where Cardialaguet et al. (8) have proved the existence of a quasi-stationary solution. These solutions are approximated in weak form using a Galerkin finite element method.

In what follows the MFG model is presented with sample solutions. The impact of reductions in federal support is explored using parameters that model the research funding system.

## Stochastic Mean Field Game Theory Model.

The MFG model gives a qualitative description of the impact of changes in government research funding on a collection of university researchers who compete for this support through a competitive peer-reviewed system. It is assumed that the number of researchers is large and constant in time so that the actions of an individual researcher are affected by the continuum distribution and not by unique individuals. It also is assumed that all researchers work to maximize their impact, measured here to be proportional to research funding, that is described by a utility function. Of course, maximizing research funding is not the sole motivation for all academic STEMM researchers who work to push back the boundaries of knowledge and create technologies that advance society. However, it is a fair motivation for a vast number of researchers who need a level of financial support to sustain their laboratory.

Following the presentation in ref. 9, the model begins with a stochastic differential equation written for the state $X_{t}$ function for an individual researcher that measures their effectiveness, as an aggregate measure of their experience, creativity, and effort:

[1] $$dX_{t}=b\left(X_{t},\alpha \left(t\right)\right)dt+\sigma dW_{t},$$

where $dW_{t}$ is additive Brownian noise with constant intensity σ. The function $b\left(X_{t},\alpha \left(t\right)\right)$ describes the evolution of the state in terms of the control parameter $\alpha \left(t\right)$ that is selected by an optimal control strategy to maximize the utility function described below. In the continuum limit of the MFG model each researcher feels the presence and behavior of others through the distribution of researchers and the SDE is rewritten in terms of a state variable x,0≤x≤1, as

[2] $$dx_{t}=b\left(x,\alpha \left(x,t\right)\right)dt+\sigma dW_{t},$$

where increasing x measures increasing effectiveness at acquiring external support. The control variable $\alpha \left(x,t\right)$ maximizes the utility function $\Phi \left(x,t,\alpha \left(x,t\right),W\left(x,t\right)\right)$ defined as

[3] $$\begin{array}{c} \Phi \left(x,t,\alpha \left(x,t\right),W\left(x,t\right)\right)\\ \equiv \int_{0}^{T}\left[L\left(x,t,W\left(t\right)\right)-S\left(\alpha \left(x,t\right)\right)\right]dt+G\left(x,W_{o}\left(x\right)\right), \end{array}$$

where W(x,t) is the distribution of researchers and $G\left(x,W_{o}\right)$ is the final value of Φ at the end of the control period 0≤t≤T. The function $L\left(x,t,W\left(x,t\right)\right)$ models the net effort of a researcher to acquire research support, and $S(\alpha \left(x,t\right)$ represents the cost of control.

The value function U(x,t) is defined as

[4] $$U\left(x,t\right)=\underset{\alpha \left(x,t\right)}{\text{sup}}E\left[\Phi \left(x,t,\alpha \left(x,t\right)\right)\right],$$

taken over all admissible control strategies $\alpha \left(x,t\right)$ and $E\left[∙\right]$ is the expected value computed using the distribution $W\left(x,t\right)$. Lasry and Lions (3–5) proved that finding $U\left(x,t\right)$ is equivalent to solving the two coupled partial differential equations formed by the Hamilton–Jacobi–Bellman (HJB) equation for U(x,t) derived from Eq. **3** and the Fokker–Planck–Kolmogorov equation (FPKE) for the probability distribution function W(x,t) derived from Eq. **2** using Ito calculus. The control trajectory $\alpha \left(x,t\right)$ and the drift velocity in the FPKE are determined from the Hamiltonian

[5] $$H\left(x,p\right)\equiv \underset{\alpha \left(x,t\right)}{\text{inf}}\left(\alpha ∙p+L(x,t,W\left(t\right))-S\left(\alpha \right)\right),$$

where p is equivalent to the momentum variable. The cost of control is modeled by the often-used quadratic expression (10, 11), $S\left(\alpha \right)=\alpha^{2}/2\gamma ,$ with constant γ, the analogy to kinetic energy. This expression also is the simplest form that guarantees the concavity (convexity in the formulation of a minimization problem) of $H\left(x,p\right)$ which is the basis for proofs of existence and uniqueness of solutions and connection to the MFG literature. Using this form the optimum control strategy determined by solving Eq. (**5**) is $\alpha \left(x,t\right)=\alpha^{*}\left(x,t\right)=b\left(x,\alpha^{*}\left(x,t\right)\right)=\gamma^{-1}\partial U/\partial x$ and is the drift velocity in the FPKE.

The analysis presented here is based on computing quasi-stationary solutions in the ergodic limit $\left(T\to \infty \right)$; it has been demonstrated (8, 12) that in this limit $W\left(x,t\right)=W_{s}\left(x\right)$ and $U\left(x,t\right)=-\lambda_{U}t+U_{s}\left(x\right)$, so that the stationary solution $\left(W_{s}\left(x\right),U_{s}\left(x\right),\lambda_{U},\lambda_{W}\right)$ satisfies the second-order differential equations:

[6a] $$\frac{\sigma^{2}}{2}\frac{d^{2}U_{s}}{{\mathrm{dx}}^{2}}+\frac{1}{2\gamma }{\left(\frac{dU_{s}}{\mathrm{dx}}\right)}^{2}+L\left(x,W_{s},N_{s}\right)=\lambda_{U}\left(HJB\right),$$

[6b] $$\frac{d}{\mathrm{dx}}\left[\frac{1}{\gamma }W_{s}\frac{dU_{s}}{\mathrm{dx}}\right]-\frac{\sigma^{2}}{2}\frac{d^{2}W_{s}}{{\mathrm{dx}}^{2}}=\lambda_{W},\int_{0}^{1}W_{s}\left(x\right)dx=1\left(FPK\right),$$

augmented by the additional constraint

[6c] $$\int_{0}^{1}U_{s}\left(x\right)dx=0,$$

that sets the constant level of $U_{s}\left(x\right)$ and determines $\lambda_{U}.The$ Lagrange multiplier $\lambda_{W}$ in Eq. **6b** is introduced so that the normalizing constraint is satisfied. Eqs. **6a–6c** are solved with the Neumann boundary conditions that the fluxes $\frac{\partial U_{s}}{\partial x}$ and $\frac{\partial W_{s}}{\partial x}$ vanish at x = 0 and x = 1. Integrating (**6b**) over $x\in \left[0,1\right]$ and applying the normalization condition and boundary conditions leads to the result $\lambda_{W}=0$, so this constant is dropped from the analysis.

The MFG model is completed by specifying a utility function $L\left(x,W_{s},N_{s}\right)$ that models competitive research funding:

[7] $$L\left(x,W_{s}\left(x\right),N_{s}\right)\equiv q\left(x,N_{s},W_{s}\left(x\right)\right)N_{s}-C\left(x,N_{s},W_{s}\left(x\right)\right),$$

where $q\left(x,N,W_{s}\left(x\right)\right)$ is the rate of acquisition of research funding from a funding level $N_{s}$ that is available for a researcher and $C\left(x,N_{s},W_{s}\left(x\right)\right)$ is the cost in effort to the researcher for acquiring and supervising the research. Using a model similar to McPike’s MFG analysis of commercial fishing (13), the rate of acquiring funding is related to the available government funding $f_{o}$ by the balance equation

[8] $$\begin{array}{c} f_{o}-E\left[q\left(x,N_{s},W_{s}\left(x\right)\right)\right]\\ =f_{o}-N_{s}\int_{0}^{1}W_{s}\left(x\right)q\left(x,N_{s},W_{s}\left(x\right)\right)dx=0. \end{array}$$

The acquisition rate and cost function are written as

[9a] $$q\left(x,N_{s},W_{s}\left(x\right)\right)=q_{o}x^{\beta_{1}}g\left(N_{s}\right)h\left(W_{s}\left(x\right)\right)p\left(x\right),$$

[9b] $$C=c_{o}x,$$

where $\left(q_{o},c_{o},\beta_{1}\right)$ are constants. In the acquisition function g($N_{s}$) models the decreased success rate on funding acquisition expected when funding is scarce (low values of $N_{s}$); here $g\left(N_{s}\right)=$$\frac{2}{\pi }\tan^{-1}\left(\delta_{N}N_{s}\right)$ with $\delta_{N}=2$. Here, $g\left(N_{s}\right)$ is chosen to vary smoothly between g(0) = 0 and g(∞)=1 with dg/dNs>0; note that g(4)>0.9. The function $h\left(W_{s}(x)\right)\equiv \frac{1}{\left(1+{\left(W_{s}(x)/W^{*}\right)}^{\beta_{2}}\right)},\beta_{2}>0$, models the increased competition for winning grants caused by a large concentration of competing researchers. The constant $W^{*}$ and the exponent $\beta_{2}$ control the magnitude of the effect for given x. When $W_{s}\left(x\right)<W^{*}$, $\beta_{2}\geq 1$ damps the effect; for $W_{s}\left(x\right)>W^{*}$, $\beta_{2}\geq 1$ magnifies the effect. The function $p\left(x\right)$ is described later in the analysis to model the skewed impact of highly effective researchers. The cost function increases with the effort/effectiveness of the researcher.

Introducing $h\left(W_{s}\right)$ gives $q\propto W_{s}^{-1}$ and adds congestion to the MFG—shifting the equilibrium away from regions of high density ($W_{s}$). Congestion in MFG theory has received considerable theoretical attention (14) because of its importance in applications and, in some formulations, has implications to uniqueness of the solutions (15).

The funding level for a researcher with effort x is

[10] $$\begin{array}{cc} S_{x}(x)\equiv q\left(x,N_{s},W_{s}\left(x\right)\right)N_{s}, & 0\leq S_{x}^{\max}<\infty \end{array},$$

where $S_{x}^{\max}=S_{x}\left(1\right)$ is the maximum funding; $S_{x}\left(x\right)$ is the natural variable for comparison of the MFG model to the analysis in ref. 2. The distribution is expressed as a function of $S_{x}$ as ${\hat{W}}_{s}\left(S_{x}\right)$ by numerically inverting Eq. (**10**). When written in terms of $S_{x}$, the expected value of $S_{x}$ satisfies $E\left[S_{x}\right]=f_{o}$.

As posed, the ergodic MFG defined by Eqs. **6**–**9** has a unique solution $\left(U\left(x\right),W\left(x\right),\lambda_{U},N_{s}\right)$. This is demonstrated by applying the technique introduced by Lasry and Lions (5) of postulating two solutions, $\left({\bar{U}}_{i}\left(x\right),{\bar{W}}_{i}\left(x\right),{\bar{\lambda }}_{U},{\bar{N}}_{s}\right)and\left({\hat{U}}_{i}\left(x\right),{\hat{W}}_{i}\left(x\right),{\hat{\lambda }}_{U},{\hat{N}}_{s}\right)$ and proving they must be identical. This is accomplished by computing the difference between Eq. (**6a**) written in terms of each solution, multiplying the result by $\left(\bar{W}\left(x\right)-\hat{W}\left(x\right)\right)$ and integrating over $x\in \left[0,1\right]$. Applying integration-by-parts, the FPKE (**6b**) expressed for each solution, the Neumann boundary conditions and the integral constraint reduces the result to

[11] $$\begin{array}{c} -\frac{2}{\gamma }\int_{0}^{1}\left[\bar{W}{\left(\frac{d\bar{U}}{\mathrm{dx}}-\frac{d\hat{U}}{\mathrm{dx}}\right)}^{2}+\hat{W}{\left(\frac{d\bar{U}}{\mathrm{dx}}-\frac{d\hat{U}}{\mathrm{dx}}\right)}^{2}\right]dx+\\ \int_{0}^{1}\left[L\left(x,\bar{W},{\bar{N}}_{s}\right)-L\left(x,\hat{W},{\hat{N}}_{s}\right)\right]\left(\bar{W}\left(x\right)-\hat{W}\left(x\right)\right)dx=0 \end{array}.$$

Because the first term in **11** is always negative, if the second term is negative definite, both terms must be zero so that $\frac{d\bar{U}}{\mathrm{dx}}=\frac{d\hat{U}}{\mathrm{dx}}$ and, by **6c**, $\bar{U}\left(x\right)=\hat{U}$(x). It also follows from (**6b**) that $\bar{W}\left(x\right)=\hat{W}\left(x\right)$ and from (**6a**) that ${\bar{\lambda }}_{U}={\hat{\lambda }}_{U}$, if ${\bar{N}}_{s}={\hat{N}}_{s}$. This last condition is proved by computing the difference between Eq. (**8**) expressed for the two postulated solutions and using the condition that dg(N)/DN>0,N>0.

Hence the solution of the MFG is unique if this condition is met and

[12] $$\begin{array}{c} \int_{0}^{1}\left[L\left(x,\bar{W},{\bar{N}}_{s}\right)-L\left(x,\hat{W},{\hat{N}}_{s}\right)\right]\\ \left(\bar{W}\left(x\right)-\hat{W}\left(x\right)\right)dx\leq 0, \end{array}$$

for any choices of the test distribution functions that are everywhere positive in the interval $\left[0,1\right]$ and satisfy the integral constraint. Using the mean value theorem for sufficiently smooth functions, Eq. (**12**) is satisfied if ∂L/∂W<0, which is true for Eq. (**7**), completing the proof of uniqueness.

The coupled boundary-value problems and the integral constraint for $\lambda_{U}$ are solved numerically by writing Eq. (**6**) in weak form and discretizing in $n_{d}-1$ linear finite elements. In this framework, the zero flux boundary conditions are incorporated naturally by neglecting boundary terms. A set of nonlinear algebraic equations $R\left(y;p\right)=0$ results that is written in terms of a solution vector $y=y\left(p\right)\epsilon R^{n_{d}+2}\left(\left\{U_{i}\right\}\epsilon R^{n_{d}},\left\{W_{i}\right\}\epsilon R^{n_{d}},\left(\lambda_{U},N_{S}\right)\epsilon R\right)$ and a vector of parameters **p.** These nonlinear equations are solved by Newton’s method with the linear algebraic equations arising at each iteration solved by LU factorization. Matlab (16) is used for the calculations reported here with $n_{d}=200$. The convergence of the Newton iterations was robust from a uniform first guess for all values of the parameters studied.

## Sample Solutions.

In the MFG model variables are expressed in terms of researchers in the distribution $W_{s}\left(x\right)$. Then the funding $N_{s}$ available for a researcher and the funding acquired, $S_{x}$, vary with federal funding $f_{o}$ according to Eqs. (**9**) as $f_{o}=q_{o}$ g($N_{s}$)E[h($W_{s}$ (x))p(x) $x^{\beta_{1}}$] $N_{s}$. As a simple base case, we consider $\beta_{2}=0$ and $\beta_{1}=p\left(x\right)=1$. Then $f_{o}={(q}_{o}/2)$ g($N_{s}$)E[*x*]N*_s_*, where E[x] is a mean value of x, and $N_{s}$ is approximately proportional to $f_{o}/q_{o}$. Note that if g($N_{s})=1,N_{s}$ can be written explicitly as $N_{s}$ = (2f_0_/q_o_)E[*x*]^−1^. Substituting this expression into the HJB gives a nonlocal (integral) coupling between $U_{s}\left(x\right)$ and $W_{s}\left(x\right)$. In this limit there is no dependence of $L\left(x,W_{s}\left(x\right),N_{s}\right)$ on $W_{s}\left(x\right)$, which for given $N_{s}$ is linear in x.

**Fig. 1.**
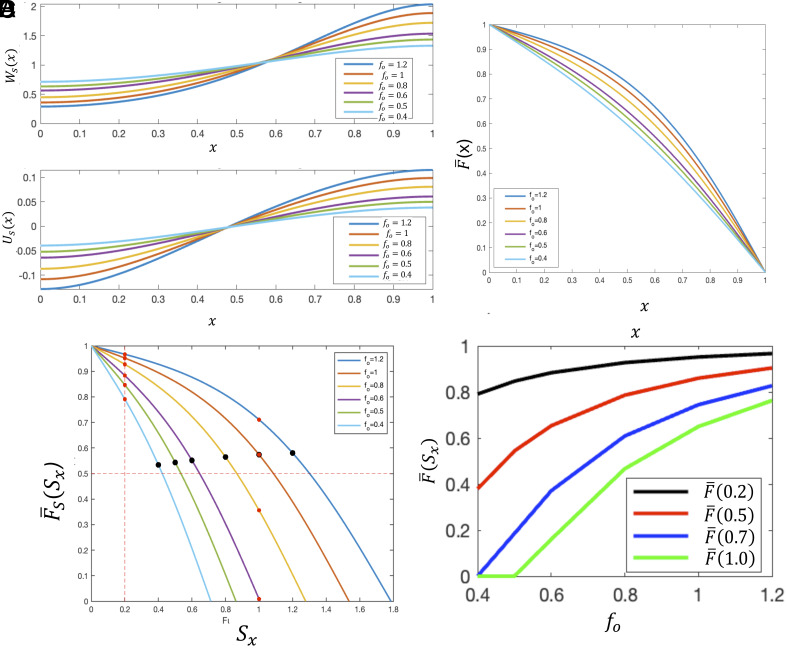
Four plots of calculations for values of $f_{o}$ and the parameter values listed in the text: (*A*) Distribution function $W_{s}\left(x\right)$ and the control function $U_{s}\left(x\right)$; (*B*) CCDF ${\bar{F}}_{x}\left(x\right)$; (*C*) CCDF ${\bar{F}}_{S}\left(S_{x}\right)$; and (*D*) values of ${\bar{F}}_{S}\left(S_{x}\right)$ for $S_{x}$ values of 0.2, 0.5, and 1.0.

Fig. 1*A* shows the distribution and control functions for this base case as a function of $f_{o},0.4\leq f_{o}\leq 1.2$. In these calculations and all that follow γ=0.25. Two features are generic to all the simulations. First, $W_{s}$(x) was largest at x = 1, demonstrating the expected bias toward more effective researchers. Also, as $f_{o}$ is decreased, the equilibrium shifted so that $W_{s}\left(1\right)$ decreased with the distribution growing for small x. In Fig. 1*B*, the mean value, E[x], shifted from 0.67 to 0.56 as $f_{o}$ decreased. The control function $U_{s}\left(x\right)$ has a similar shape to $W_{s}$(x).

The complementary cumulative distribution function (CCDF), ${\bar{F}}_{x}\left(x\right)\equiv 1-\int_{0}^{x}W_{s}\left(x\right)dx$, gives the fraction of researchers with effort above x. As shown in Fig. 1*B*, ${\bar{F}}_{x}\left(x\right)$ decreased with decreasing $f_{o}$. Expressing ${\bar{F}}_{x}\left(x\right)$ in terms of $S_{x}\left(x\right)$ gives ${\bar{F}}_{S}\left(S_{x}\right)$ as plotted in Fig. 1*C*. For given $f_{o}$, researchers were distributed with a fraction having $S_{x}\left(x\right)$ > $f_{o}$ up to a maximum $S_{x}^{\max}$ that depended on $f_{o}$. For $f_{o}=1,S_{x}^{\max}=1.54$, corresponding to ${\bar{F}}_{x}\left(1\right)=0$ or $-{\overset{-}{F}}_{S}\left(S_{x}^{\max}\right)=0$.

The mean values E[*S_x_*] for given $f_{o}$ are marked by black dots in Fig. 1*C* and decreased slightly with decreasing $f_{o}$. For these parameters, E[*S_x_*] was less than the median $S_{x}^{\mathrm{med}}$, given by ${\bar{F}}_{S}\left(S_{x}^{\mathrm{med}}\right)=0.5$, indicating a negatively skewed distribution. The decrease in $S_{x}$ with decreasing $f_{o}$ was more dramatic. Taking $f_{o}=1$, as the level before funding cuts, ${\bar{F}}_{S}\left(1\right)$ decreased from 0.57 to 0.01 for $f_{o}=0.5$. Essentially no researchers had the level of funding available they had before the decrease. The variation of ${\bar{F}}_{S}$ with $f_{o}$ is shown in Fig. 1*D*.

The base case assumed linear variation of the utility function with x. The shape of the distribution function $W_{s}\left(S_{x}\right)$ computed by differentiation of 1- ${\bar{F}}_{S}\left(S_{x}\right)$ was sigmoidal beginning with $S_{x}=0$ and had a maximum at $S_{x}^{\max}$. $W_{s}\left(S_{x}\right)$ approached a uniform distribution for small values of $f_{o}$, as implied by the linearity of ${\bar{F}}_{S}\left(S_{x}\right)$.

Results are presented below for parameter values of the utility function tuned to better model the current funding system.

## Impact of Decreased Funding.

The analysis is connected to research university faculty funding levels by using the USN&WR 2022 survey of national universities that reported the average expenditures per faculty for engineering schools. For the 146 Carnegie R1 universities with engineering schools the average per faculty expenditures per year ranged from $ 112,000 to $ 1,407,000 with a mean of $ 502,000. These expenditures were from all sources including federal, state, business, foundations, and the university. Both direct and indirect expenditures are included. It was not possible to separate these sources; however, for all research in R1’s, approximately 57 percent of total research came from the federal government, according to the NSF HERD survey (17). Using this fraction, the average federal support for engineering faculty is estimated as $ 286,000 annually.

In ref. 2, faculty with research per year less than $100,000 annually of external support were characterized as research inactive—having inadequate funding to support a viable research program; this level corresponded to approximately 20 percent of the mean expenditure from the USN&WR survey. Here, ${\bar{F}}_{S}\left(0.2\right)$ was used to designate the demarcation between active and inactive researchers.

Also, typically a small fraction of the faculty raised a disproportionate amount of the funding, giving the appearance of a Pareto-like distribution of funding, in which the mean is significantly larger than the median (2). This effect was simulated by adding the function p(x) to the acquisition function, Eq. (**9a**), where $p\left(x\right)\equiv (1+\sigma_{x}\exp(\theta_{x}\left(x-1\right)/\delta_{x})$, with $\delta_{x}\ll 1$ and $\theta_{x}=\left(1\right)$, to increase $q\left(x,N,W\left(x\right)\right)$ near x = 1. Note that $p\left(0\right)≅1$, $p\left(1\right)=1+\sigma_{x}$ and $p\left(1-\delta_{x}\right)=\left(1+\sigma_{x}e^{-\theta_{x}}\right)≅1$ so that adding p(x) significantly boosted researchers with effectiveness in the range $\left(1-\delta_{x}\right)\leq x\leq 1$. Increasing the parameter $\beta_{1}>1$ in Eq. **9a** also damped funding of researchers with lower effectiveness.

**Fig. 2.**
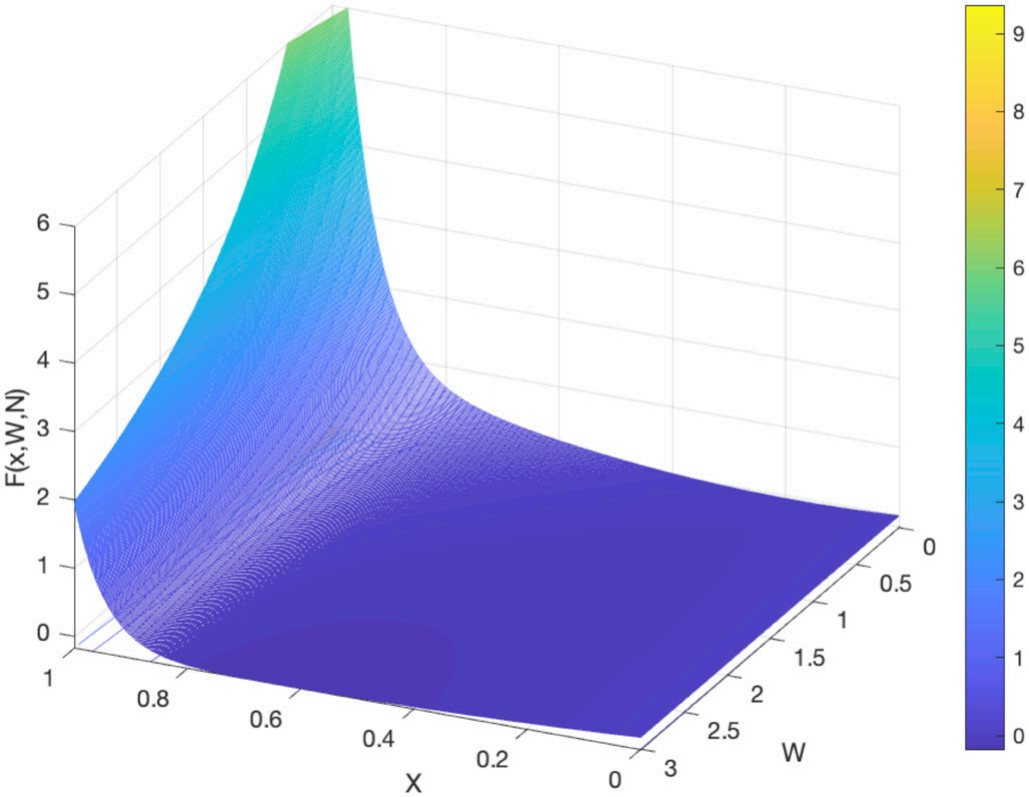
The utility function $F\left(x,W_{s}\left(x\right),1\right)$ for the parameters listed in the text constructed to be indicative of the competition for funding.

**Fig. 3.**
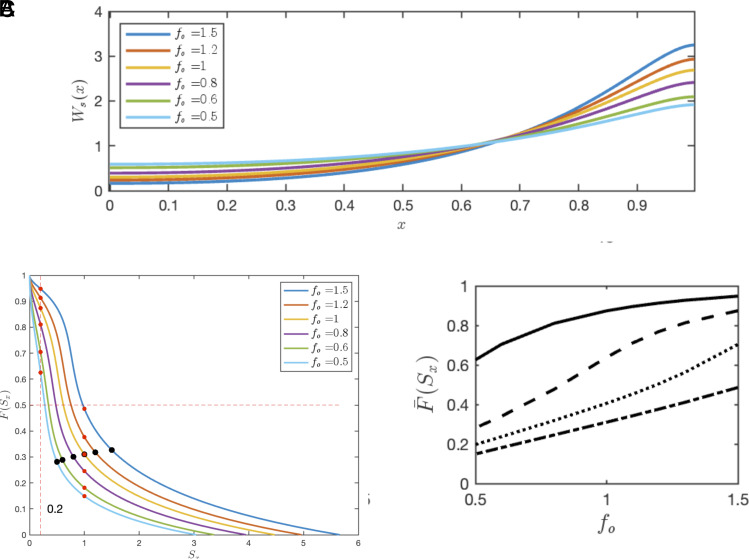
Three plots of calculations for values of $f_{o}$ and the utility function shown in Fig. 3: (*A*) the Distribution function $W_{s}\left(x\right)$; (*B*) CCDF ${\bar{F}}_{S}\left(S_{x}\right)$; and (*C*) values of ${\bar{F}}_{S}\left(S_{x}\right)$ for $S_{x}$ values of 0.2 (—), 0.5 (---), 0.7 (…), and 1.0.(−•−).

Solutions were computed for the parameter set $\left(\beta_{1},\beta_{2,}q_{o},c_{o}\right)=\left(2,1,2,0.5\right)$. The congestion effect $h\left(W_{s}\right)$ was included with $W^{*}=1$. Finally, the parameters in p(x) were set as $\left(\delta_{x},\sigma_{x},\theta_{x}\right)=\left(0.3,6,5\right)$. The utility function $L\left(x,W_{s}\left(x\right),N_{s}=1\right)$ for these parameters is shown in Fig. 2. The function reached high levels (set by $\sigma_{x}$) for $W_{s}\left(x\right)<1\;and\;x>1-\delta_{x}$ and then declined for lower x and greater $W_{s}\left(x\right)$. The distribution $W_{s}\left(x\right)$ computed for these parameters and for $0\leq f_{o}\leq 1.5$ is shown as Fig. 3*A*. The bias toward researchers with x near 1 flattened the distribution for smaller x and increased $W_{s}\left(x\right)$ as x→1. With varying $f_{o}$, $W_{s}\left(x\right)$ pivots about a point close to $x=\delta_{x}=0.3$, where p(x) began to increase the utility function.

**Fig. 4.**
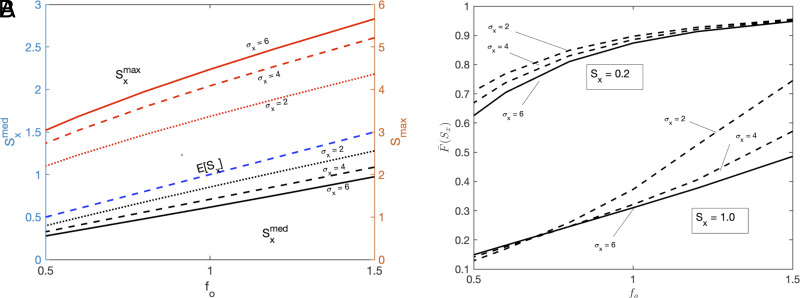
Variation of four solution measures with increasing the bias toward high performing researchers by increasing $\sigma_{x}$: (*A*) $E\left[S_{x}\right]\;\text{and}\;S_{x}^{\mathrm{med}}$ as a function of $f_{o}$; and (*B*) ${\bar{F}}_{S}\left(0.2\right)$ and ${\bar{F}}_{S}\left(01.0\right)$ as a function of $f_{o}$. Other parameters are for the base case shown in Figs. 2 and 3.

The ccdf ${\bar{F}}_{S}\left(S_{x}\right)$ is shown in Fig. 3*B* for these parameter values. Most striking is the distribution’s tail that grows as $f_{o}$ is increased; $S_{x}^{\max}=5.66$, for $f_{o}=1.5$, up from $S_{x}^{\max}=1.07$ for $f_{o}=0.5$. The long tail was caused by shifting funding to researchers with x near 1 and resulted in the median E[*S_x_*] becoming greater than the median, as seen by comparing the black dots with ${\bar{F}}_{S}\left(S_{x}^{\mathrm{med}}\right)=0.5$ for given $f_{o}$. This shift also is plotted in Fig. 4.

The impact of decreasing $f_{o}$ on the fraction of researchers with adequate funding is obvious from Fig. 3*B*. Taking $S_{x}=1$ (the base-case funding level) as a reference, decreasing $f_{o}$ to 0.5 decreased ${\bar{F}}_{S}\left(1\right)$ from 0.38 for $f_{o}=1$ to only 0.15 for $f_{o}=0.5$; only 15 percent of the faculty have funding that was equivalent to the original funding level. The variation of four values of ${\bar{F}}_{S}\left(S_{x}\right)$ with $f_{o}$ is shown in Fig. 3*C*. Perhaps most significant was the decrease in ${\bar{F}}_{S}\left(0.2\right)$, the value of $S_{x}$ associated with the demarcation between active and inactive researchers; for the same decrease in $f_{o}$, ${\bar{F}}_{S}\left(0.2\right)$ varied from 0.87 to 0.63. Almost a quarter of the researchers that were previously research active were no longer.

These results are the major conclusion of this analysis. Two points need to be addressed; the sensitivity of the results to the selected parameter values and the relatively small number of inactive faculty computed for $f_{o}=1$, compared to the empirical data for an engineering faculty (2) that suggests that the fraction ${\bar{F}}_{S}\left(0.2\right)$ may be much larger than the 0.13 predicted here.

## Parametric Sensitivity.

The sensitivity of the results described above was investigated through a series of calculations varying key parameters. Typical calculations are shown in Fig. 4 for varying $\sigma_{x}$ and $f_{o}$. Increasing $\sigma_{x}$ increased the impact of the bias to high performing researchers, increasing $S_{x}^{\max}$ (Fig. 4*A*) and decreased the funding for the median researcher as more resources move into the tail of the distribution. The distribution became more skewed with an increasing difference between the mean and the median. These effects remain with decreasing $f_{o}$; for $f_{o}=0.5,S_{x}^{\max}$ is three times E[*S_x_*]. The shifting of funding to researchers in the tail of the distribution also is apparent in the values of ${\bar{F}}_{S}\left(S_{x}\right)$ for $S_{x}=0.2$ and $S_{x}=1.0$ plotted in Fig. 4*B* for increasing $\sigma_{x}$. Although the tail of the distribution ${\bar{F}}_{S}\left(S_{x}\right)$ is long, it did not have the form of a Pareto distribution, ${S_{x}}^{-\alpha }$; it was not linear on a log–log plot. This is not unexpected as Pareto distributions typically arise from multiplicative stochastic processes (18, 19) compared to the additive SDE (1) that is the basis for the MFG.

## Inactive Researchers: A Two Population MFG Model.

The MFG model above assumes that all researchers are executing a similar strategy for maximizing the utility function, i.e. working to acquire external research support. This is not the case in research universities where there are faculty members who have stopped (or never began) attempting to support a research program—they are really not in the game. Not considering these inactive researchers may account for the relatively high fraction of active researchers (87.4 percent) predicted for $f_{o}=1$.

A more accurate description of the research faculty is a mixture model of two populations; (A) active researchers computed from the MFG model, and (I) inactive researchers who are not participating. Assuming there is a distribution of each, the CCDF of the mixture ${\bar{F}}_{m}\left(S_{x}\right)$ is written as

[13] $$\begin{array}{c} {\bar{F}}_{m}\left(S_{x}\right)=\left(1-\alpha_{r}\right){\bar{F}}_{A}\left(S_{x}\right)+\alpha_{r}{\bar{F}}_{I}\left(S_{x}\right), \end{array}$$

where $\alpha_{r}$ is the fraction of inactive researchers in (I) and ${\bar{F}}_{A}\left(S_{x}\right)={\bar{F}}_{S}\left(S_{x}\right)$. Examining the curves in Fig. 1*B*, computed with a utility function essentially proportional to x, suggests that as $f_{o}$ is decreased toward zero, ${\bar{F}}_{I}\left(x\right)\to 1-x$. In this limit $F\left(x,W_{s}\left(x\right),N_{s}\right)\to \varepsilon x,\varepsilon \ll 1$, and the CCDF for researchers in (I) is ${\bar{F}}_{I}\left(S_{x}\right)≅\varepsilon^{-1}S_{x}$, for $0\leq S_{x}\leq \varepsilon$, and 0 for $S_{x}\geq \varepsilon$. Then for $S_{x}>\varepsilon$, ${\bar{F}}_{m}\left(S_{x}\right)=\left(1-\alpha_{r}\right){\bar{F}}_{A}\left(S_{x}\right)$. Thus, the effect of the inactive researchers is to lower the distribution ${\bar{F}}_{m}\left(S_{x}\right)$ by $\left(1-\alpha_{r}\right)$ for all values of $S_{x}$ except in a small region near x=0. This conclusion can be rigorously justified by asymptotic solution of the MFG model in the limit ε≪1 and implies that the true fraction of inactive researchers is ${\bar{F}}_{m}\left(0.2\right)=\left(1-\alpha_{r}\right){\bar{F}}_{S}\left(0.2\right)$ so that if $\alpha_{r}=0.2$, the inactive fraction including those attempting to support a research effort is 1-0.8∙0.87=0.3 for $f_{o}$ = 1 and 0.54 for $f_{o}$ = 0.5. These results are in approximate agreement with those in ref. 2.

## Discussion

The faculty in research universities across the United States rely on meritocratically administered peer-reviewed external research funding in order to fuel innovation in science, medicine, and engineering. Mean field game theory and the Nash equilibria computed here model the dynamics of the system when perturbed either by changes in funding or, potentially, by introduced biases (20). The results clearly indicate the dramatic impact of funding decreases on the distribution of funding. If the 40 percent decrease originally proposed by the Administration in 2025 came into effect—either in 1 y or over time—there would be a large fraction of previously research active faculty who will have little external funding. The model predicts that, if available funding is halved, over 50 percent of researchers would have less than the minimal needed level of support. The number of active faculty may become too low to maintain the vibrancy of the research environment and doctoral programs in many academic departments and whole institutions (2). The decline could lead to aggregating active researchers in “institutes,” where university resources can be focused.

The predictions here beg the question of what a research university should do to be effective in a less supportive federal environment. It is not a plan to either hope that the funding tide turns or simply let the research enterprise shrink organically. For decades most universities have taken the approach of letting “a thousand flowers bloom” in their research garden. The fear going forward is that this approach could simply lead to a smaller and less impactful research enterprise.

I and others (2, 21) believe that to survive, a research university will need to be intentional about its research strategy, organizing it as a central mission of the university and making hard decisions about emphasizing disciplines and topics where the institution can compete effectively for external support. This is best done with centralized, collaborative leadership and financial management. Even with such systems, very difficult and unpopular decisions will be needed about where to focus resources, knowing that going-forward, sustaining the area could require considerable university support. Research support that will be weighed against the institutional pressures for cost control, promoting student access and success, and embracing AI. It will be difficult to justify propping up the research enterprise at the expense of a low graduation rate.

The predictions of the MFG model assumes that all participants are pursuing the same strategy, in this case working to acquire external funding for their research. It is quite possible that a prolonged period of reduced funding will result in some researchers “dropping out” and shifting to the permanently inactive category and, over time, more faculty being hired only to teach and not to do research. The result would be fewer faculty in the game and an increase in available funding per faculty $f_{o}$ without additional federal support; however, it implies faculty split between two tracks. In most universities, this may be in inevitable and divisive result of decreased external funding.

Because the computations are of ergodic states, transients associated with moving from one value of $f_{o}$ to another are ignored. It is likely that a large reduction in funding will occur as a series of cuts that are best modeled by stepwise reductions in $f_{o}$. Calculating these transients requires solution of the forward/backward time-dependent versions of Eq. (**6**) setting the time horizon for the HJBE to the length of the annual funding cycle. The ergodic solutions are only approximations to these intermediate states.

Finally, the analysis for a second population of inactive researchers is a prelude to the analysis of a MFG represented by two or more populations with different strategies and abilities for optimizing their funding while competing for one pot of funding. The models might describe a scenario where the competition between the two-populations is represented by the negative influence of one population on the cost function of the other or by one population having superior expertise for acquiring funding. It is well established (22, 23) that under such conditions, two population MFG models can lead to segregation of agents with those in one population essentially winning over the other. More ominously, we should worry that a two-population model may also be appropriate if the Administration injects bias into the awarding of grants based on anything other than the merit of the research; then the second population would be the advantaged researchers.

## Data Availability

There are no data underlying this work.
